# Characterization of Infant Formulae Marketed in Italy and Virulence Potential of *Bacillus cereus* Isolates

**DOI:** 10.3390/foods15030536

**Published:** 2026-02-03

**Authors:** Viviana Fusi, Simone Stella, Emilia Ghelardi, Francesco Celandroni, Cristian Bernardi, Maria Filippa Addis, Clara Locatelli, Chistian Scarano, Francesca Piras, Giuliana Siddi, Erica Tirloni

**Affiliations:** 1 Department of Veterinary Medicine and Animal Sciences, University of Milan, Via dell’Università 6, 26900 Lodi, Italy; viviana.fusi@unimi.it (V.F.); cristian.bernardi@unimi.it (C.B.); filippa.addis@unimi.it (M.F.A.); clara.locatelli@unimi.it (C.L.); 2 Department of Translational Research and New Technologies in Medicine and Surgery, University of Pisa, Via San Zeno 37, 56127 Pisa, Italy; emilia.ghelardi@unipi.it (E.G.); francesco.celandroni@unipi.it (F.C.); 3 Research Center Nutraceuticals and Food for Health-Nutrafood, University of Pisa, 56128 Pisa, Italy; 4 Department of Veterinary Medicine, University of Sassari, Via Vienna 2, 07100 Sassari, Italy; scarano@uniss.it (C.S.); fpiras@uniss.it (F.P.); g.siddi1@phd.uniss.it (G.S.)

**Keywords:** microbiota, powdered infant formulae, follow-on formulae, formulae for special medical purposes, contamination

## Abstract

This study aimed to evaluate the microbiological quality and safety of powdered formulae intended for infant consumption on the Italian market. A total of 83 samples, including 23 infant formulae (PIF), 42 follow-on formulae (FOF), and 18 formulae for special medical purposes (SMPs), were taken between 2023 and 2024. Low total viable counts were highlighted with all SMP samples, 87.0% of PIF samples and 97.6% of FOF samples being compliant with the threshold set by the Code of Hygienic Practice for Powdered Formulae for Infants and Young Children (2.70 Log CFU/g). High contamination levels (>4 Log CFU/g) were found exclusively in PIF (8.7%) and FOF samples (2.4%). Considering potential pathogenic bacteria, the presence of *Listeria monocytogenes*, *Yersinia enterocolitica*, *Salmonella* spp., *Bacillus cereus*, and *Cronobacter sakazakii* was investigated. Enumeration of *Bacillus cereus*, *Staphylococcus aureus*, and *Clostridia* was also performed. Only presumptive *B. cereus* was detected (37 samples, and in 3 samples was enumerated with counts equal to 1 Log CFU/g). A total of 42 presumptive *B. cereus* isolates were tested for the production of hemolysin BL, phosphatidylcholine-specific phospholipase C, proteases, and for the presence of chromosomal toxin-encoding genes, showing a relevant prevalence of virulence factors and highlighting a potential concern for infants. The antimicrobial resistance pattern of the isolates showed high resistance rates to β-lactams and a moderate resistance to erythromycin. A chemical–physical characterization of the formulae was also performed showing high heterogeneity in terms of pH, Aw, and concentration of organic acids. The results obtained provide useful information for monitoring the potential exposition of infants to microbial populations and to evaluate the safety of the products available on the market.

## 1. Introduction

When breastfeeding is not possible or sufficient, substitute preparations made from powdered milk can be used. There are several types of products available on the market, categorized based on age, such as powdered infant formula (PIF) and follow-on formula (FOF), or based on particular nutritional needs, such as formulae for special medical purposes (SMPs). Despite high hygiene standards, these products may still become contaminated with microorganisms (spores, thermophilic bacteria) that pose health risks to infants, including, in some cases, susceptible subjects such as premature, low birth weight, or immunocompromised individuals [1].

Production follows three possible processes: the wet-mix process, in which the ingredients are heat-treated together before drying; the dry-mix process, in which the ingredients are heat-treated individually and then blended; and the combined process, which incorporates aspects of both processes [2]. In any of these processes, contamination of the powdered milk can occur due to the presence of thermoduric and spore-forming bacteria surviving the treatments, as well as contamination along the production line until packaging. Moreover, reconstitution and handling by caregivers represent critical points for potential contamination [3]. The temperatures used during spray drying (at least 60–80 °C) [4], although effective in preserving the nutritional properties of various ingredients and reducing microbial loads, do not eliminate all potential spoilage and pathogenic microorganisms. In particular, spore-forming bacteria, including *Bacillus* and *Clostridium*, may survive in these conditions [5,6,7].

Several international studies have confirmed the sporadic presence of pathogenic bacteria and have enumerated spoilage organisms in commercial powdered formula products [8,9,10,11,12,13,14,15,16,17,18,19,20,21,22,23,24,25,26]. The survival and persistence of spore-forming microorganisms during the shelf life has required special attention: contamination by mold [8], *Clostridium* spp. [9,10,11], and *Bacillus cereus* [12,13,14] compromises both the safety and quality of these products. Under favorable conditions, spores can germinate, leading to product spoilage, toxin production, and contamination of food with potentially infective bacteria, posing a serious risk to the underdeveloped digestive and immune systems of newborns [7,8,9,10,11,12,13,14]. *B. cereus*, in particular, is considered the etiological agent of emetic or diarrheal syndromes: diarrheal syndrome is caused by the ingestion of foods contaminated by *B. cereus* vegetative cells that, once ingested, grow in the gut and actively secrete toxins; while emetic syndrome is an intoxication due to the ingestion of the preformed toxin cereulide produced by strains growing as contaminants in foods, mostly rice, pasta, milk, or dairy products. The infectious dose for *B. cereus* realistically ranges between 30 and 200 CFU/mL of milk [27]. In order to prevent safety concerns, EC Regulation 1447/2007, amending EC Reg. 2073/2005, set process hygiene criteria for dried infant formulae and dried dietary foods intended for special medical purposes for infants below six months of age, with thresholds of 50 and 5 × 10^2^ CFU/g for presumptive *B. cereus* [16].

Although not spore-formers, *Cronobacter sakazakii* and *Salmonella* spp. also play a critical role in these products, as they have been linked to multiple outbreaks and severe neonatal infections. In particular, *C. sakazakii* contamination has resulted in multiple fatalities and cases of permanent injury, underscoring the severe impact of this pathogen in neonates. *C. sakazakii* and *Salmonella* outbreaks linked to PIF have been reported in several countries with several fatalities and permanent sequelae in the population [17,18,19,20,21,22,23,24,25].

Despite growing global awareness, microbiological studies focusing on PIF, FOF, and SMP products sold on the Italian market remain limited and often fragmented, with each investigation targeting only a specific subset of parameters, such as lactic acid bacteria, *Cronobacter* spp., or *B. cereus* [26,27,28,29]. This lack of data limit an accurate risk assessment and the development of targeted monitoring and control strategies. Moreover, very little data on the antimicrobial resistance of isolates from milk powder sold in Italy are available [30].

In the present study, microbiological quality and safety parameters were investigated in PIF, FOF, and SMP formulae sold in Italian supermarkets. A wide range of spoilage and potential pathogenic bacteria were considered, with bacteria isolated from the formulae that were submitted to the assessment for antibiotic resistance and for the evaluation of the presence of virulent genes. Finally, a physicochemical characterization, useful to support microbiological findings and to better understand product variability, was also performed.

## 2. Materials and Methods

A total of 83 powdered milk products were included in this study: 23 PIF, 42 FOF, and 18 SMP products (including SMP products intended for allergy, for colic constipation, and for regurgitation problems). These were selected based on their diverse formulation from 10 different brands (in order to give a representative picture of the market), and purchased at different retailers in Northern Italy during two sampling sessions between December 2023 and November 2024 (Supplementary File S1). Intact product packs were transported to the lab and analyzed immediately.

### 2.1. Microbiological Analyses

Microbiological analyses were performed singularly as follows. Briefly, 10–15 g of product (dry powder) were aseptically taken from intact packs (weighing from 400 to 800 g), diluted 10-fold in a chilled sterile diluent solution (0.85% NaCl and 0.1% peptone) in sterile bags (Interscience, Saint-Nom-la-Bretèche, France), and homogenized for 60 s in a Stomacher™ 400 Circulator (Seward Medical, London, UK). Subsequently, appropriate 10-fold dilutions of the homogenates were prepared in chilled saline (ISO 6887-1) [31]. The following parameters were enumerated by spreading aliquots on solid culture media: total mesophilic bacterial count (ISO 4833) [32], anaerobic bacterial count (Plate Count Agar, PCA; plates were incubated in hermetic jars [AnaeroJar™, Oxoid, Basingstoke, UK] with anaerobiosis generators [AnaeroGen™, Oxoid, Basingstoke, UK] at 30 °C for 48 h), lactic acid bacteria (LAB) (ISO 15214) [33]; plates were incubated at 30 °C in anaerobic conditions as described above), *Pseudomonas* spp. (ISO 13720) [34], *Enterobacteriaceae* (ISO 21528-2) [35], *Escherichia coli* (ISO 16649-1) [36], *Clostridia* (ISO 15212-2 [37], *Enterococcus* spp. (Slanetz–Bartley Agar in aerobic conditions at 37 °C for 48 h), yeast and mold (ISO 21527-1) [38], coagulase-positive Staphylococci (CPS) (ISO 6888-1) [39], and *B. cereus* (PEMBA agar, Scharlab, Barcelona, Spain, incubated at 30 °C for 48 h).

For the detection of *Salmonella* spp. and *C. sakazakii*, 25 g of powdered formula were aseptically taken from intact packs, homogenized in 225 mL of Buffered Peptone Water (BPW; Scharlab, Barcelona, Spain), and incubated at 37 °C for 24 h for pre-enrichment [40]; the analyses were then performed following the ISO 6579-1 method [41] for *Salmonella* spp. and the ISO 22964 [42] method for *C. sakazakii.* As low *B. cereus* counts were expected, an enrichment method was also applied. In particular, 25 g of powdered formula were aseptically taken from intact packs, homogenized in 225 mL of Buffered Peptone Water (BPW; Scharlab, Barcelona, Spain), incubated at 30 °C for 48 h, and stroked onto PEMBA agar.

To evaluate the presence of *Listeria monocytogenes* and other *Listeria* spp., the ISO 11290-1method was applied [43]; for the detection of *Listeria monocytogenes*, RAPID’L.mono agar (Bio-Rad Laboratories, Hercules, CA, USA) was inoculated, while Palcam agar (Scharlab, Barcellona, Spain) was used for the detection of *Listeria* spp. Finally, for the detection of *Yersinia enterocolitica*, the ISO 10273:2017 method was applied [44].

When present on the plates, up to five colonies per plate were selected for identification, based on distinct morphological characteristics to ensure representation of bacterial diversity. Each selected colony was isolated on Columbia Blood Agar with Sheep Blood (Thermo Fisher Scientific, Milan, Italy), and was subsequently identified using matrix-assisted laser desorption ionization–time of flight mass spectrometry (MALDI-TOF MS) with the MALDI Biotyper^®^ System (MBT) (Billerica, MA, USA) [45]. A small amount of material from an isolated colony was spread onto one well of the target plate with a toothpick, overlaid with 1 μL of 70% formic acid and let dry. Then, 1 μL of α-cyano-4-hydroxycinnamic acid in 50% acetonitrile and 2.5% trifluoroacetic acid (Bruker Daltonik GmbH, Bremen, Germany) was deposited on the well and let dry. Spectra were acquired with a microflex™ mass spectrometer (Bruker Daltonik GmbH, Bremen, Germany) in positive mode, equipped with the subtyping module for *Listeria* spp. identification. Bacterial Test Standard (Bruker Daltonik GmbH, Bremen, Germany) was used to calibrate the instrument. Spectra were interpreted with the MBT Compass^®^ 4.1 database against the spectrum library version 2023. The thresholds for genus-level identification and species-level identification were log (score) of ≥1.7 and ≥2.0, respectively, according to the manufacturer’s instructions. After identification, colonies were maintained at −80 °C in Microbank Cryogenic vials for subsequent investigation (Pro-Lab Diagnostics UK, Bromborough, Wirral, UK).

### 2.2. Phenotypic Detection of Virulence Factors

*Bacillus cereus* isolates were submitted to phenotypic detection of virulence factors. Hemolysin BL (HBL) production was evaluated by streaking bacterial cultures onto Columbia Blood Agar supplemented with 5% sheep blood (Oxoid). Plates were incubated at 30 °C for 18 h, and HBL production was assessed by observing the distinctive zone of hemolysis around colonies [46]. Phosphatidylcholine-specific phospholipase C (PC-PLC) activity was assessed by streaking bacterial cultures onto the Blood Agar Base (Oxoid, UK) and plates containing 0.15% L-α-phosphatidylcholine (Sigma-Aldrich, Milan, Italy) were incorporated into the medium. PC-PLC activity appeared as a precipitation halo surrounding colonies. Proteolytic activity was tested by growing the isolates on 1.2% agar plates containing 1.5% skim milk (Oxoid, UK) at 37 °C for 18 h [47]; protease production was indicated by clear zones surrounding the colonies. All tests were performed in triplicate on different days to ensure consistency.

### 2.3. Molecular Detection of Toxin Genes

Genomic DNA from *B. cereus* isolates was extracted according to established protocols [48]. Briefly, cells were harvested from liquid cultures at the late exponential growth phase, washed with a TES buffer (5 mM EDTA, 50 mM NaCl, 30 mM Tris-HCl, pH 8.0), and suspended in 12 mL of TES. An amount of 20 mg lysozyme and 5 mg RNase were added, the samples were incubated at 37 °C for 40 min, and 8% Triton X-100 (2.1 mL) and 10 mg mL^−1^ proteinase K (0.6 mL) were added. After incubation at 37 °C for 1 h, 2.5 mL CTAB/NaCl solution (10% CTAB; 0.7 M NaCl) was added, and the samples were incubated for an additional 10 min at 65 °C. DNA purification was performed with chloroform isoamyl alcohol (24:1), extracted by phenol, and precipitated with isopropanol. To determine the presence of toxin-encoding genes in *B. cereus*, a conventional PCR was performed on the extracted genomic DNA. Target genes are indicated in Table 1; amplification protocols were adopted from previously validated methodologies [48,49].

### 2.4. Antimicrobial Resistance

*Bacillus cereus* isolates were submitted to antimicrobial susceptibility testing by broth microdilution using the Sensititre™ automated system and the GPALL1F plate (Thermo Fisher Scientific, Milan, Italy), according to the manufacturer’s instructions. Bacterial suspensions were adjusted to 0.5 McFarland in sterile water, 10 µL were inoculated into Mueller–Hinton Broth (Thermo Fisher Scientific, Milan, Italy), and 50 µL of the resulting inoculum were dispensed into each well. Plates were incubated aerobically at 35 °C for 24 h. The minimum inhibitory concentration (MIC) was defined as the lowest concentration (µg/mL) of each antimicrobial that inhibited visible bacterial growth. Internal QC wells were included, and *Staphylococcus aureus* ATCC 29213 served as the quality control strain.

The antimicrobials tested, along with their detection ranges, were as follows: ampicillin (0.12–8), penicillin (0.06–8), oxacillin + 2% NaCl (0.25–4), chloramphenicol (2–16), clindamycin (0.5–2), erythromycin (0.25–4), daptomycin (0.5–4), rifampin (0.5–4), gentamicin (2–16), streptomycin (500), linezolid (1–8), vancomycin (0.25–32), trimethoprim/sulfamethoxazole (0.5/9.5–4/76), tetracycline (2–16), tigecycline (0.03–0.5), ciprofloxacin (1–2), levofloxacin (0.25–4), moxifloxacin (0.25–4), nitrofurantoin (32–64), and quinupristin/dalfopristin (0.5–4). Among these, for antimicrobials with available EUCAST breakpoints, the MIC values were interpreted according to the corresponding EUCAST clinical guidelines (version 15.0; EUCAST, 2025) [50]. For antibiotics lacking EUCAST breakpoints for *Bacillus* spp., MIC values were instead compared with susceptibility thresholds reported in the scientific literature for *B. cereus*. Isolates were classified as multidrug-resistant (MDR) if they were resistant to at least one agent in three or more antimicrobial classes [51]. When evaluating multidrug resistance, intrinsic resistance traits known to occur in *B. cereus*, such as resistance to β-lactam antibiotics [52,53], were not considered as acquired resistance and were excluded from MDR classification, in accordance with Magiorakos et al. [51].

### 2.5. Physicochemical Analyses

Moisture content was determined according to Bradley and Vanderwarn [54] by placing 3 ± 0.25 g of formula in an oven at 100 °C for 16.5 h. Water activity (Aw) was also measured using Rotronic Hygromer Aw-DIO (Bassersdorf, Switzerland). Each sample was reconstituted with distilled water following the manufacturer’s instructions, and the pH was measured using a pH meter (Amel Instruments, Milan, Italy).

Concentrations of organic acids were determined by HPLC (from an adapted version of [55], reported in Tirloni et al. [56]). One gram of formula sample was dissolved in 10 mL of mobile phase (0.005 N H_2_SO_4_ prepared by diluting reagent grade sulfuric acid (Sigma-Aldrich, St. Louis, MO, USA) with distilled water, filtering through a 0.45 μm RC membrane filter (Scharlab, Barcelona, Spain)); then the samples were centrifuged at 14,000× *g* for 15 min and the supernatant was filtered through a 0.2 μm regenerated cellulose (RC) membrane (Scharlab, Barcelona, Spain). The analysis was performed using an HPLC system consisting of two PU-1580 HPLC pump (Jasco, Cremella, Italy), a 717 plus autosampler, and a 481 UV detector (Waters, Milford, MA, USA) set at 210 nm. The isocratic separation was carried out at a flow rate of 0.5 mL/min and a temperature of 40 °C on a Rezex ROA (Phenomenex, Torrance, CA, USA) 300 mm × 7.8 mm, 8 μm.

External commercial standards (Sigma-Aldrich, St. Louis, MO, USA) were used for identification and quantification of acetic, citric, lactic, propionic, and butyric acids. For a determination of the linearity of each target organic acid, eight concentration points in triplicate were used to calculate the regression line and the coefficients of determination (R2: 0.9997, 0.9998, 0.9990, 0.9998, and 0.9994 for acetic, citric, lactic, propionic, and butyric acid, respectively). The limit of detection (LOD: 13, 31, 25, 15, and 17 mg/kg for acetic, citric, lactic, propionic, and butyric acid, respectively) and limit of quantification (LOQ: 23, 83, 69, 44, and 50 mg/kg for acetic, lactic, citric, propionic, and butyric acid, respectively) were calculated by the signal-to-noise approach.

### 2.6. Statistical Analyses

The data obtained from microbiological and chemical–physical analyses were submitted to statistical analyses, with the aim to evaluate the differences between product typologies (PIF, FOF, SMPs) and intended use (SMPs for allergy, colic constipation, or regurgitation). For microbiological data, a Fisher exact test was applied, considering the prevalences of detection (*B. cereus*) or of detectable counts (total mesophilic aerobic count, total anaerobic count, LAB, mold, and *B. cereus*); a significance threshold of 0.05 was applied. Data obtained from chemical–physical analyses (pH, Aw, moisture %, and concentration of organic acids) were analyzed by one-way two tail ANOVA. A significance threshold of 0.05 was applied.

## 3. Results and Discussion

### 3.1. Microbiological Characterization

In the present study, a total of 83 powdered milk formulae for infants sold on the Italian market were characterized for microbiological and physicochemical characteristics. Counts of total mesophilic bacteria (TVC), anaerobic bacteria, and LAB are reported in Table 2 and Table 3. Generally, the products evaluated in the present research showed a satisfactory microbiological quality: most samples showed very low total mesophilic counts, and counts were detectable (≥2 Log CFU/g) in a small percentage of samples (17%, 13%, and 10% in SMP, PIF, and FOF samples, respectively, without a statistically significant difference). High contamination levels (>4 Log CFU/g) were found exclusively in PIF (8.7%) and FOF samples (2.4%), with no SMP samples exceeding this level. In all the samples with high TVC, LAB were also detected in high counts, representing all the microbial population: this was confirmed by a MALDI-TOF identification of isolates from the media used from TVC and LAB counts (Supplementary File S2). The threshold for a good microbiological quality of the products (2.70 Log CFU/g), as indicated by the Code of Hygienic Practice for Powdered Formulae for Infants and Young Children [57], was respected by all SMP samples, by 87.0% of PIF samples, and by 97.6% of FOF samples. One PIF sample showed a count between 2.70 and 3.70 Log CFU/g, falling within a range considered to be marginally acceptable. However, two PIF samples and one FOF sample exceeded the limit of 3.70 Log CFU/g, which corresponds to defective quality (none of these four powdered formulae samples contained voluntarily added microorganisms like LAB). The findings of our study were consistent with the data reported in the literature, with a large proportion of powdered milk products on the international market that fall within acceptable microbiological limits [58,59]. In the study by Iversen and Forsythe [58], 78 of 82 PIF samples from the UK, Europe, Asia, Africa, and the USA had TVC below or equal to 2 Log CFU/g. Koseki et al. [59] found that 102 out of 136 FOF from Brazil, Indonesia, Jordan, Korea, Malaysia, Portugal, and the UK had TVC below 2 Log CFU/g, while 7 were defective with TVC above 4 Log CFU/g.

These typology of products may represent a potential biological risk for infants, as they are not fully sterilized during the production process and as it is also inevitable that bacterial contamination occurs. It is also very important to highlight the bacterial community members and their role, although the products do not offer adequate conditions for spore germination and, on the contrary, rehydration and feeding stages may offer a favorable environment. The total bacterial count may represent, in this sense, a vertical and horizontal expansion. In the case of vertical expansion, the total bacterial count may be a very useful indicator and could be extended from enumeration targets to risk factors, including toxins and spoilage indicators, but can be also an indicator of antibiotic resistance providers and bacterial communities interacting with potential pathogenic bacteria [60,61].

The microflora of the products characterized in our study showed the predominant presence of *Bacillus* spp. (*B. licheniformis*, *B. subtilis*, *B. sonorensis*, and *B. cereus*), with the sporadic isolation of other thermoduric species (*Limosilactobacillus fermentum*, *Staphylococcus warneri*, *Klebsiella aerogenes*, and *Weizmannia ginsengihumi*). These bacteria are not frequently detected in raw milk; but, in dried formulae, they are able to persist and can be found in several stages the opportunity to grow during the manufacturing process. This may be due to their better ability to survive compared to the natural microflora present in raw milk (e.g., *Pseudomonas*). Moreover, in the form of spores, they resist and concentrate in the powders during the processes. The empirical evidence that *Bacillus* spp. can be the cause of foodborne outbreaks is not yet clear and needs the enrollment of advanced epidemiological tools in outbreak investigations. In any case, this predominance should also be considered as a novel risk factor for the transmission of antibiotic resistance genes among the bacterial community of these products, as gene transfer among *Bacillus* spp. has already been described [60,61].

Regarding anaerobic bacterial counts, most samples showed very low levels, without significant differences among the product typologies. Additionally, LAB counts were below the detection limit of 2 Log CFU/g in all SMP samples, as well as in most PIF and FOF samples (in this case, no statistically significant differences were detected). High counts (≥5 Log CFU/g) were detected in 4 PIF samples and 6 FOF samples. MALDI-TOF-MS allowed for the identification of *Limosilactobacillus fermentum* in 3 PIF and 4 FOF samples, all from the same brand, while *Lactobacillus reuteri* was identified in 1 PIF and 2 FOF samples, all belonging to another brand (Supplementary File S2). These LAB species are known for their probiotic activity, their ability to resist pasteurization processes, and are frequently used in human nutrition [62,63] and added to various formulations, such as infant formulae; their presence in the analyzed products could presumably be referred to as an intentional addition, even if not specifically reported in the label. All samples with high anaerobic counts also showed high levels of LAB, suggesting the overlap of microbial counts (indeed the same species were identified among isolates taken from the two media; Supplementary File S2).

The presence of mold was sporadic in the products analyzed (without differences among the typologies) and characterized by very low counts. However, a single PIF sample (4.4%) showed a count exceeding 4 Log CFU/g. All mold isolates were identified as *Penicillium* spp., a common environmental contaminant. The low fungal contamination level was in agreement with previous reports [8]. Despite the sporadic occurrence of mold in PIF, our results underscore the importance of monitoring for occasional contamination that could compromise product quality and safety, also taking into account the potential production of mycotoxins. Further studies are needed in this field to fill the gap.

*Pseudomonas* spp., *Enterobacteriaceae*, *E. coli*, *Enterococcus* spp., yeasts, and coagulase-positive Staphylococci were never detected in counts ≥ 2 Log CFU/g. The literature data indicate the possible presence of *Pseudomonas* spp. (*P. fulva* and *P. putida* were isolated by Cawthorn et al. [64]) and the relatively frequent detection of *Enterobacteriaceae* (in particular *E. coli*) with detectable counts [27,28,64,65,66], Enterococci (40% of PIF and FOF samples) and coagulase-positive Staphylococci [8]. The presence of *Clostridium* spp. spores, previously reported as a possible contaminant [9], was not confirmed in our study. Considering potential pathogenic bacteria, *Salmonella* spp., *C. sakazakii*, *Listeria* spp., *L. monocytogenes*, and *Y. enterocolitica* were never detected in the products. The potential contamination of infant formulae by pathogenic bacteria must be continuously considered by the producers. The absence of samples harboring *Salmonella* spp. and *C. sakazakii* in our study was in agreement with previous Italian data [29], but other studies have described the occasional isolation of these pathogens from infant formulae [67,68,69,70]. Although *C. sakazakii* was not recovered from unopened products, we should be aware that, in previous studies from the USA, this microorganism was found to be present in water containers and open products, confirming the possible environmental route of contamination in the domestic kitchen. In a previous study, feeding utensils, like spoons, surfaces present in the kitchens, as well as pacifiers were found to be positive for the presence of this pathogen, confirming the crucial role of the consumer’s handling in the domestic kitchen for spread of the pathogens. Consumers are responsible for prevention of the transmission of pathogens by adopting hygienic practices during the reconstitution and somministration of PIF. According to the WHO/FAO, in order to prevent adverse effects in the preparation of infant formula for bottle-feeding at home, the use of hot water at 70 °C or higher is recommended, and the infant formula should be discarded within 2 h after being prepared. Moreover, a previous web survey highlighted that some Japanese mothers did not comply with the recommended guidelines suggested for the reconstitution and somministration of PIF, laying the product until 2 h at room temperature, thereby endangering infant health [71].

Detectable counts of presumptive *B. cereus* were found in 3.61% of the analyzed products: in two PIF samples (corresponding to two batches of the same product, 1 Log CFU/g) and in one SMP sample (1 Log CFU/g). However, *B. cereus* was detected in 44.6% of the samples after subculturing, suggesting its widespread presence in powdered formulae; although a higher prevalence was detected in FOF samples, no significant differences among FOF, PIF, and SMP samples were found. Our data comply with the limits reported in Reg. 1441/2007 [16], with all the analyzed samples showing counts below the limit of 50 CFU/g.

This prevalence based on quantification is closer to that reported by Di Pinto et al. [29], who found *B. cereus* in 5 of 60 Italian PIF samples (8.3%), with contamination levels below 6 Log CFU/g, and lower than that reported by Ibrahim et al. [12], who found *B. cereus* in 26.7% of samples, with a higher mean count of 1.3 Log CFU/g. Similarly, Becker et al. [72] reported *B. cereus* counts in 55.8% of 206 samples (PIF, FOF, and SMP samples combined), with levels ranging from <1 to 2.78 Log CFU/g. Lesley et al. [73] detected *B. cereus* in 5 of 12 powdered formula samples (41.7%), with levels ranging from <0.47 to >3.04 Log MPN/g, though notably, none of their PIF samples were positive. Compared to these studies, our low enumeration rate suggests a relatively limited bacterial load in the tested products, even if enrichment revealed a higher prevalence. The low counts detected in our samples may indicate a possible persistence in the environment and may likely point to scarce sanitation procedures during production. The recurrence of quantifiable levels in multiple batches of the same product raises concerns about potential biofilm formation on processing surfaces, a known survival strategy of *B. cereus* [74], and should be deepened in terms of virulence factor and antimicrobial resistance pattern.

In this sense, a total of 42 presumptive *B. cereus sensu lato* colonies were isolated from subsequent tests: 3 were isolated from enumeration plates, while the other colonies were isolated from the streaks of subculturing. Their presence in this type of product may constitute a concern for infant health and a serious risk for these consumers [75].

### 3.2. Detection of B. cereus Virulence Factors

**Figure 1:**
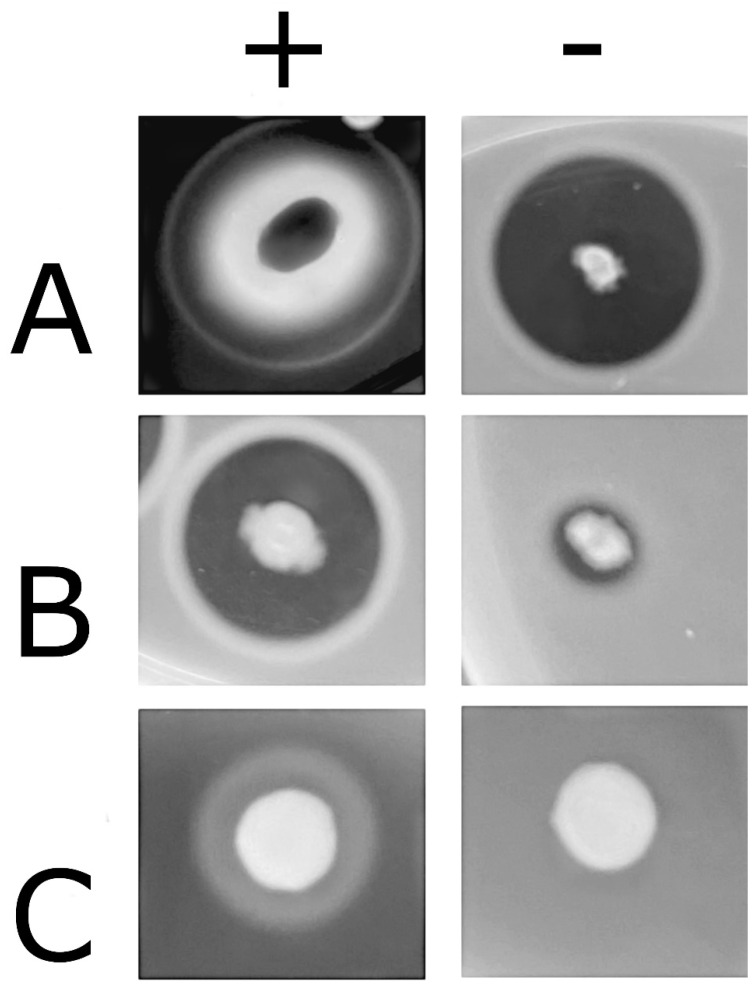
Phenotypic production of different *Bacillus cereus* virulence factors: HBL, protease, and PC-PLC. Representative samples of (**A**) HBL production (+, discontinuous halo of hemolysis; -, hemolysis due to other endolysins); (**B**) Protease secretion (+, halo of secretion; -, halo of secretion close to the lower limit of detection); and (**C**) PC-PLC precipitation halo.

The ability of all *B. cereus* isolates to produce virulence proteins and enzymes, such as proteases, phospholipases, hemolysin BL, and cereulide (Figure 1 and Table 4), was evaluated. All the strains were able to produce proteases, with 2 strains close to the lower limit of detection, while 21 strains (50%) secreted PC-PLC and 28 (67%) secreted tripartite hemolysin HBL. These results agree with previous data showing a very high rate of proteases, and a variable rate of HBL production (ranging from 20% to 90%) among *B. cereus* strains, although herein we found a lower rate of PC-PLC production (67% versus 92%) [76]. Regarding other virulence factors, no specific assays are available. The most represented toxin gene was *sph* (present in 97.7% of the strains). The prevalence of *bceT* obtained in this study (45.2%) is lower if compared to the prevalence reported in Tirloni et al. [77] for strains isolated from fresh and short-ripened old cheeses on the Italian market. A lower prevalence was also reported by Zhao et al. [78]. The presence of the three genes *nheA*, *nheB*, and *nheC* was revealed in 76.2% of the isolates, a prevalence that is in line with previous data [29,77,78]. The *entFM* gene was present in 64.3% of the strains, a prevalence similar to that described in the literature for other dairy products [77,78]. Lastly, the *cytK* gene, encoding *CytK*, was only present in 7.1% of the strains: this result was not in agreement with what found by Di Pinto et al. [29], who highlighted 100% presence of *cytK* in 12 strains isolated from infant milk powder in Italy. The *ces* gene was present in 28.6% of the isolates; *ces*-positive strains are not frequently reported in food samples, very often their occurrence is related to fatalities due to contamination [79,80,81]. Differently from our study, in the study by Sadek et al. [82], *cytK* was carried by 95.5% of milk-based infant food with fruit, vegetables, honey, rice, and wheat, while *nhe* was present in 71.1% of the samples.

The presence of a cereulide-producing strain in PIF raises concern, as the toxin may be pre-formed in the reconstituted PIF. This toxin is very scarcely present among *B. cereus* strains isolated from dairy products [77], but it should be carefully focused as it may lead to severe consequence, such as damage to the liver and multi-organ failure. A total of seven isolates expressed simultaneously all toxigenic genes except one (*ces*, *sph*, *entFM*, *entS*, *nheA*, *nheB*, *nheC*, *bceT*, and *cytK*), while three isolates expressed simultaneously three toxigenic genes (*nheA*, *nheB*, *nheC*, *bceT*, and *cytK*).

The observation that only two strains were able to produce all the enzymes and toxins and to possess all the genes encoding virulence factors except one (*ces* or *cytK*), should not let our guard drop concerning the presence of *B. cereus* in the sample formulae. Indeed, the virulence potential of this germ is multifactorial, and pathogenicity does not require the contextual presence of all virulence factors. As reported by the EFSA in 2016 [83], cells or spores of *B. cereus* in a count above 10^4^ CFU/g are sufficient to produce diarrheal toxins in the human gut and intestine. According to Food Standards Australia New Zealand [84], *B. cereus* might reach an infectious dose within 4 h if stored at room temperature starting from a count of 100 CFU/g. Moreover, according to Lewin et al. [15], an infectious dose for *B. cereus* for a particularly susceptible population (extremely weak neonates) realistically ranges between 30 and 200 CFU/mL of milk. Our data showed a general safe situation, with a maximum count of 10 CFU/g (giving a low final concentration in the reconstituted milk, in the range of 1–2 CFU/mL); although the initial count is low, great care should be paid to the management of milk leftovers in order to avoid *B. cereus* growth.

#### Antibiotic Resistance

All 42 *B. cereus* isolates were analyzed for their antibiotic resistance profiles (Table 5 and Table 6). As expected, a high proportion of isolates exhibited resistance to β-lactam antibiotics. This phenotype is considered intrinsic to *B. cereus* [52,53] and is not necessarily related to the presence of acquired resistance mechanisms. Considering only non-intrinsic resistance, according to the EUCAST 2025 criteria, 13 of 42 isolates (30.9%) exhibited resistance to at least one of the tested antibiotics. Specifically, resistance was detected in 1/42 isolates (2.4%) to ciprofloxacin, 1/42 isolates (2.4%) to clindamycin, and 11/42 isolates (26.2%) to erythromycin. All resistant isolates were resistant to a single antibiotic class only; consequently, no multidrug-resistant (MDR) phenotypes were identified in this study. Overall, the MIC distribution revealed marked differences in antimicrobial activity across the tested agents (Table 6). As expected, *B. cereus* isolates exhibited uniformly high MICs to β-lactam antibiotics (ampicillin, penicillin, and oxacillin), reflecting their intrinsic resistance. By contrast, levofloxacin, moxifloxacin, vancomycin, and linezolid showed low MIC values, with MIC_50_ and MIC_90_ consistently within the susceptible range. For ciprofloxacin, most isolates showed MIC values below the lowest quantifiable concentration on the testing panel, although its MIC distribution could not be fully resolved due to panel limitations. Moderate variability was observed for chloramphenicol, clindamycin, erythromycin, tetracycline, and trimethoprim/sulfamethoxazole, which displayed broader MIC distributions with small subsets of less susceptible isolates. Nitrofurantoin MICs were uniformly ≤32 mg/L, but no further stratification was possible. Overall, the isolates showed full susceptibility to several clinically relevant agents, including linezolid and vancomycin, and nearly complete susceptibility to clindamycin (97.6%). Resistance to erythromycin was also relatively low (26.2%). A low resistance rate was also observed for ciprofloxacin; however, this result should be interpreted cautiously due to the limited MIC range of the testing panel, which may have led to an underestimation of resistance. Further analyses using testing panels covering the full EUCAST MIC range are needed to more accurately characterize ciprofloxacin susceptibility. The evaluation of antibiotic resistance in the *B. cereus* population, combined with the detection of virulence genes, is particularly important, as the onset of multidrug resistance in *B. cereus* strains is of clinical concern, especially considering the potential to cause foodborne illness with symptoms ranging from diarrhea and vomiting to more severe complications in infants, like fulminant liver failure, rhabdomyolysis, and metabolic acidosis [85,86], which could be combined with limited treatment options. Our data were consistent with those highlighted by Zhao et al. [78], Wang et al. [87], and Shlegelova et al. [88], who detected high resistance rates to ampicillin and penicillin, and a low rate for ciprofloxacin and clindamycin. Nonetheless, Zhao et al. reported a lower resistance rate to erythromycin (16.7%). Italian data about *B. cereus* strains obtained from various food sources (including dairy products) generally confirmed our results, although a lower resistance rate for erythromycin (6.8%) and, on the opposite, a higher resistance rate for clindamycin (19.5%) were detected [53]. These data indicate a general low resistance burden for *B. cereus*; nevertheless, Ibrahim et al. [12] reported high resistance levels in *B. cereus* isolates from infant formula in Egypt. The issue of antimicrobial resistance is significant for public health, underlying the need for control measures and monitoring in food production to reduce antimicrobial resistance. If we consider the combination of antibiotic resistance and virulence factors harbored by the *B. cereus* isolates, our data showed that all the antibiotic-resistant strains produced protease, whereas only five and four strains produced PC-PLC and HBL, respectively. Considering the genes encoding virulence factors, all these isolates carried *sph*, whereas none carried *cytK*, four strains carried *ces* and/or the complete enterotoxin-encoding pattern, and just one showed a not quite complete virulence pattern (excluding PC-PLC and *cytK*).

### 3.3. Physicochemical Characterization

The pH, Aw, and moisture content were evaluated for SMP, PIF, and FOF products, revealing some differences among the product types, as summarized in Table 7 and Supplementary File S3.

SMP samples showed an average pH of 6.34, significantly lower than both PIF (6.55; *p* < 0.05) and FOF samples (6.66; *p* < 0.01). Median pH values followed the same trend. The pH range was also narrower in SMP samples (5.80–6.74) compared to the FOF samples (5.97–7.24), indicating more consistent formulation among SMP products. Moreover, significantly lower pH values (*p* < 0.01) were detected in SMP samples intended for allergy problems (6.03 ± 0.18) compared to the other two typologies (6.40 ± 0.43 and 6.39 ± 0.10 for SMP-colic constipation and SMP-regurgitation, respectively). Having a lower pH in infant formula may accelerate and favor the protein digestive process in infants, as the pH of the infant’s stomach is higher after a meal and pepsin activity is limited or inhibited, thereby limiting gastric protein digestion.

The Aw was extremely low and constant across all categories, as expected for powdered products. SMP samples had a slightly lower average Aw (0.214) compared to PIF (0.226) and FOF samples (0.222). However, PIF samples showed the widest Aw range (0.133–0.317), suggesting more variability in moisture control during processing or packaging. Generally, due to the low Aw, none of the analyzed samples resulted in a substrate for microbial growth (Aw < 0.32). These conditions may vary during the reconstitution of the product, and thus should be reconsidered during this stage [89].

Moisture content followed a similar pattern. SMP samples had slightly but significantly higher humidity (mean value of 2.56%) compared to PIF and FOF samples (2.17% and 2.15%, respectively). SMP samples also exhibited the narrowest humidity range, again reflecting greater consistency.

The concentrations of citric, lactic, acetic, propionic, and butyric acids were quantified in SMP, PIF, and FOF samples, revealing inter-product variability (Table 8). Citric acid was the most abundant organic acid across all product types. The FOF samples showed the highest average concentration (9062 mg/kg), followed by PIF (6870 mg/kg) and SMP samples (4784 mg/kg); these differences were statistically significant (*p* < 0.05 vs. PIF samples and *p* < 0.01 vs. SMP samples). Among SMP samples, SMP-allergy showed the lowest mean value (2336 mg/kg) compared to SMP-colic constipation (4761 mg/kg) and SMP-regurgitation (5616 mg/kg). Variability in citric acid content was notable, particularly in FOF samples, which exhibited a wide range from 4537 to 21,770 mg/kg. Lactic acid presence varied substantially (also if not significantly) among the product types. SMP samples had the highest average lactic acid content, though with a high standard deviation, indicating notable sample-to-sample variation. In particular, a low mean value was detected in SMP-allergy (427 mg/kg) compared to the other two typologies (1463 and 1955 mg/kg in SMP-colic constipation and SMP-regurgitation, respectively). Acetic acid was present at low levels in the analyzed samples; indeed, in many samples, it resulted in <LOQ. SMP samples showed the highest average content, followed by PIF and FOF samples. However, SMP samples also displayed the greatest variability. For lactic acid, lower values were detected in SMP-allergy samples (63 mg/kg) compared to the other samples (140 and 177 mg/kg in SMP-colic constipation and SMP-regurgitation, respectively). Propionic acid content was present in almost all the analyzed samples; its concentrations was significantly lower in SMP samples compared to PIF (*p* < 0.05) and FOF samples (*p* < 0.01), with SMP-allergy samples showing significantly lower values (mean of 147 mg/kg) compared to SMP-colic constipation (2645 mg/kg, *p* < 0.05) and SMP-regurgitation (3592 mg/kg, *p* < 0.01). Butyric acid content was also detected but not in all the samples; concentration was limited with significantly lower concentrations in SMP samples compared to FOF samples (*p* < 0.05); SMP-allergy and SMP-colic constipation values (average of 3364 and 1387 mg/kg, respectively) were significantly higher compared to SMP-regurgitation ones (147 mg/kg). No correlation was found between high LAB counts and organic acid concentrations, indicating the absence of an evident production by the microbiota. The presence of lactic, propionic, and butyric acids may be related to their natural presence in milk, the main component of infant food. Acetic acid concentration was variable among the considered brands, but the amounts were generally low. Acidification of infant formulae with organic acid (in particular propionic, acetic, and lactic acid) has been reported as an effective system to contrast the proliferation of pathogenic bacteria [90,91]. In the labels of the 83 samples considered, only citric acid was indicated. This was matched by analyses where only this organic acid was found in considerable amounts in most of the analyzed samples It should be noted that potassium or sodium citrate are generally inserted as a component in all the analyzed samples. The presence of citric acid alone results in weak bactericidal activity; when in combination with caprylic acid, however, it results in more effective activity [92].

It is also important to evaluate the substrate of growth of the microorganisms found in powdered milk formulae for infants. Concerning physicochemical characteristics, SMP products displayed more uniform profiles, while PIF and FOF products showed wider variability, particularly in water activity. These differences may reflect variations in formulation, manufacturing processes, or requirements for shelf-life and stability. The high degree of consistency observed in both microbiological and physicochemical parameters in SMP products likely reflects the stringent quality standards and the need for uniformity, given that these formulae are intended for a particularly vulnerable group of infants.

## 4. Conclusions

Although the overall results are comforting in terms of microbiological quality and, in many aspects, safety, the prevalence, virulence potential, and antibiotic resistance profiles of *B. cereus* isolates raise important concerns. In a future study, intravariability among batches and brands will be useful to better understand the distribution among batches of the microbial populations. The findings obtained from this study underscore the need for the continuous monitoring of powdered formula products, particularly considering the vulnerability of the target population. Future studies will focus on assessing the growth potential of these *B. cereus* strains in reconstituted formula, which can provide a favorable environment for their replication and on the capability to produce biofilm.

## Figures and Tables

**Table 1:** Detection of target genes for virulence factors on *B. cereus* isolates.

Gene	Specific Primers Pairs for Identification	
Sphingomyelinase (*sph*)	Ph1: cgtgccgatttaattggggc	Ph2: caatgttttaaacatggatgcg
Enterotoxin BceT (*bceT*)	ETF: ttacattaccaggacgtgctt	ETR: tgtttgtgattgtaattcagg
Enterotoxin S (*entS*)	TY123: ggtttagcagcagcttctgtagctggcg	TY125: gtttcgttagatacagcagaaccacc
Phosphatidyl inositol—specific phospholipase C (*plcA*)	PC105: cgctatcaatggaccatgg	PC106: ggactattccatgctgtacc
Enterotoxin FM (*entFM*)	ENTA: atgaaaaaagtaatttgcagg	ENTB: ttagtatgcttttgtgtaacc
Cytotoxin K (*cytK*)	F2: aacagatatcggtcaaaatgc	R7: cgtgcatctgtttcatgagg
Non-hemolytic enterotoxin components (*nheA*)	344S: tacgctaaggaggggca	843A: gtttttattgcttcatcggct
Non-hemolytic enterotoxin components (*nheB*)	1500S: ctatcagcacttatggcag	2269A: actcctagcggtgttcc
Non-hemolytic enterotoxin components (*nheC*)	2820S: cggtagtgattgctggg	3401A: cagcattcgtacttgccaa
Cereulide (*ces*)	cesF1: ggtgacacacattatcatataaggtg	cesR2: gtaagcgaacctgtctgtaacaaca

**Table 2:** Distribution (%) of the main microorganisms in SMP, PIF, and FOF samples.

Parameter	Count Range (Log CFU/g)	SMP (%)	PIF (%)	FOF (%)
Total mesophilic bacteria	<2	83.3	87.0	90.5
	2–3	16.7	0.0	7.1
	3–4	0.0	4.3	0.0
	>4	0.0	8.7	2.4
Anaerobic bacteria	<2	100.0	82.6	83.3
	2–3	0.0	0.0	4.8
	3–4	0.0	4.3	2.4
	4–5	0.0	4.3	0.0
	≥6	0.0	8.7	9.5
LAB	<2	100.0	82.6	85.7
	2–3	0.0	0.0	0.0
	3–4	0.0	0.0	0.0
	4–5	0.0	0.0	0.0
	≥5	0.0	17.4	14.3

Limit of detection LOD = 2 Log CFU/g.

**Table 3:** Microbial counts (Log CFU/g) for total mesophilic bacteria, anaerobic bacteria, and lactic acid bacteria in SMP, PIF, and FOF samples, excluding counts < LOD.

Category	SMP			PIF			FOF		
Parameter	Total Mesophilic Bacteria (3) *	Anaerobic Bacteria	LAB	Total Mesophilic Bacteria (3) *	Anaerobic Bacteria (5) *	LAB (4) *	Total Mesophilic Bacteria (4) *	Anaerobic Bacteria (7) *	LAB (6) *
Mean ± DS	2.36 ± 0.10	<LOD	<LOD	5.45 ± 1.69	4.52 ± 2.23	6.51 ± 0.30	3.39 ± 2.59	4.98 ± 2.05	6.50 ± 0.45
Min	2.30	<LOD	<LOD	3.50	1.30	6.06	2.00	2.30	6.14
Max	2.48	<LOD	<LOD	6.46	6.65	6.73	7.26	7.27	7.37

* number of samples with enumerable counts (>LOD = 2 Log CFU/g).

**Table 4:** Detection of virulence potential including secretion of virulence factors and presence of toxin-encoding genes in *B. cereus* isolates.

Strain	Phenotypic Assays			Gene Amplification Assays								
	Proteases	PC-PLC	HBL	*ces*	*sph*	*entFM*	*entS*	*nheA*	*nheB*	*nheC*	*bceT*	*cytK*
3	+	+	-	-	+	+	+	+	+	+	+	+
6	+	+	-	-	+	+	+	+	+	+	-	-
7	+	-	+	-	+	+	+	+	+	+	+	-
7b	+	-	+	+	+	+	+	+	+	+	+	-
9	+	+	+	+	+	+	+	+	+	+	+	-
10	+	+	-	+	+	+	+	+	+	+	+	-
12	+	+	+	-	+	+	-	+	-	+	-	-
14	+	-	+	-	+	+	+	+	+	+	+	-
17	+	+	+	-	+	+	+	+	+	+	-	-
18	+	-	+	-	+	+	+	+	+	+	+	-
20	+/-	-	+	-	+	-	-	+	+	+	+	-
22	+	-	+	+	-	-	+	-	-	-	-	-
23	+	-	+	+	+	+	+	+	+	+	+	-
24	+	-	+	-	+	+	+	+	+	+	+	-
33	+	+	+	-	+	+	-	-	-	+	-	-
35a	+	-	+	-	+	+	+	+	+	+	+	+
35b	+	+	-	-	+	-	-	+	+	+	-	-
36	+	+	-	-	+	-	-	-	+	+	-	-
37	+	+	+	-	+	+	+	+	+	+	+	+
39	+	-	-	-	+	+	+	+	+	+	-	-
40	+	+	-	-	+	-	-	+	+	-	-	-
41	+	-	+	-	+	+	-	+	+	+	-	-
42	+	-	+	+	+	+	-	+	+	+	-	-
43	+	+	-	+	+	+	-	+	+	+	+	-
49a	+	-	+	-	+	+	+	+	+	+	+	-
49b *	+	-	+	-	+	+	+	+	+	+	+	-
54	+	+	-	+	+	+	+	+	+	+	+	-
55	+	-	+	-	+	+	+	+	+	+	+	-
57	+	+	+	-	+	-	+	+	+	+	+	-
58	+	-	+	-	+	+	+	+	+	+	+	-
64	+	+	-	-	+	-	-	-	-	-	-	-
65	+	-	+	+	+	+	+	+	+	+	-	-
66	+	-	-	-	+	-	-	+	+	+	-	-
68	+	+	+	+	+	-	+	+	-	+	-	-
73	+	+	+	-	+	-	+	+	+	+	-	-
74a	+/-	+	+	-	+	-	+	-	-	-	-	-
74b *	+	-	-	-	+	-	+	-	-	-	-	-
75a	+	-	-	+	+	+	-	+	+	+	-	-
75b *	+	-	-	+	+	+	-	+	+	+	-	-
77	+	+	+	-	+	-	-	-	-	+	-	-
78	+	+	+	-	+	-	+	+	+	+	-	-
79	+	+	+	-	+	-	+	+	+	+	-	-

* Isolated from enumeration. “+/-” indicates close to the limit of detection; “+” indicates the secretion of virulence factor or presence of the corresponding gene.

**Table 5:** Antibiotic susceptibility profiles of 42 *B. cereus* isolates susceptible (S) and resistant (R) according to EUCAST 2025 breakpoints.

Antibiotic	S (%)	R (%)
Ciprofloxacin	41 (97.6)	1 (2.4)
Clindamycin	41 (97.6)	1 (2.4)
Erythromycin	31 (73.8)	11 (26.2)
Levofloxacin	42 (100.0)	0
Linezolid	42 (100.0)	0
Vancomycin	42 (100.0)	0

**Table 6:** Distribution (%) of MIC values, MIC_50_, MIC_90_, and EUCAST (2025) resistance breakpoints for the antimicrobial agents tested against *Bacillus cereus* isolates.

Antibiotic Agent	Distribution (%) of MICs (mg/L)											Eucast Breakpoint R > (mg/L)	MIC_50_ (mg/L)	MIC_90_ (mg/L)
	0.03	0.06	0.12	0.25	0.5	1	2	4	8	16	32			
Ampicillin			2.4			9.5	33.3	14.3	40.5					
Chloramphenicol							74.4	23.3	2.3				2	4
Ciprofloxacin					97.6		2.4					0.5		
Clindamycin				97.6			2.4					1	0.25	0.25
Daptomycin					25.6	9.3	30.2	34.9					2	4
Erythromycin				69.7	4.7	11.6	9.3	4.7				0.5	0.25	2
Gentamicin							100						2	2
Levofloxacin				79.5	18.2	2.3						1	0.25	0.5
Linezolid						100						2	1	1
Moxifloxacin				93.2	4.5	2.3							0.25	0.25
Nitrofurantoin											100			
Oxacillin + 2% NaCl				7	2.3			90.7					4	4
Penicillin		2.3				2.3	23.3	20.9	51.2				8	8
Quinupristin/Dalfopristin					14	83.7	2.3						1	1
Rifampin					100								0.5	0.5
Streptomycin 1000 ug/mL														
Tetracycline							79.1	14	2.3	4.6			2	4
Tigecycline	2.3	23.3	65.1	9.3									0.12	0.12
Trimethoprim/Sulfamethoxazole					60.5	7	2.3	30.2					0.5	4
Vancomycin					21.4	76.2	2.4					2	1	1

Values represent the proportion of isolates inhibited at each MIC dilution. Ciprofloxacin and nitrofurantoin MIC values are omitted due to testing panel limitations.

**Table 7:** Physical-chemical parameters of SMP, PIF, and FOF samples.

		SMP			PIF			FOF	
	pH	Aw	Moisture (%)	pH	Aw	Moisture (%)	pH	Aw	Moisture (%)
Average ± DS	6.34 ^b^ ± 0.29	0.2135 ± 0.0539	2.56 ^a^ ± 0.45	6.55 ^a^ ± 0.26	0.2256 ± 0.0699	2.17 ^b^ ± 0.62	6.66 ^a^ ± 0.23	0.2223 ± 0.0571	2.15 ^b^ ± 0.47
Median	6.35	0.2012	2.53	6.56	0.2564	2.22	6.67	0.2090	2.12
Min	5.80	0.1428	1.94	5.89	0.1327	0.94	5.97	0.1252	1.21
Max	6.74	0.3234	3.45	7.04	0.3166	3.09	7.24	0.3196	3.10

^a,b^ = *p* < 0.05.

**Table 8:** Concentration of organic acids in powdered formulae (mg/Kg).

	Acid	Citric	Lactic	Acetic	Propionic	Butyric
SMP	Average	4784 ^B^ (18/18)	2610 (18/18)	782 * (3/18)	3313 *^,b,B^ (14/18)	1902 *^,b^ (10/18)
	DS	2070	1059	860	1.402	1330
PIF	Average	6870 ^b^ (23/23)	2529 * (9/23)	582 * (2/23)	4177 *^,a^ (21/23)	1255 * (14/23)
	DS	1667	1130	358	1.335	1093
FOF	Average	9062 ^a,A^ (42/42)	2329 * (16/42)	248 * (2/42)	4371 *^,A^ (40/42)	987 *^,a^ (17/42)
	DS	3579	755	4	1793	460

* Average obtained from samples > LOQ; the number of samples are reported in brackets > LOQ. ^a,b^ = *p* < 0.05, ^A,B^ = *p* < 0.01.

## Data Availability

The original contributions presented in this study are included in the article/Supplementary Materials. Further inquiries can be directed to the corresponding authors.

## Supplementary Materials

The following supporting information can be downloaded at: https://www.mdpi.com/article/10.3390/foods15030536/s1, Supplementary File S1: Powdered formulae arranged by brand, category and age of consumpion; Supplementary File S2: Microbial characterization and isolates identification; Supplementary File S3: Chemical-physical characterization.
