# Supplementary material for: Characterization of Infant Formulae Marketed in Italy and Virulence Potential of Bacillus cereus Isolates
Source: Foods. 2026 Feb 3;15(3):536. doi: 10.3390/foods15030536 (PMC12897385; doi:10.3390/foods15030536)
Supplement: Supplementary file 1 [file foods-15-00536-s001.zip › foods-4074325-supplementary.pdf]

Supplementary file S1

Powdered formulae arranged by brand, category and age of consumpion.

- ✓ *powdered infant formula (PIF)*
- ✓ *follow-on formula (FOF)*
- ✓ *based on particular nutritional needs, e.g. formulae for special medical purposes (SMP)*

#	Brand	Category - Indication	Age (months)
1	A	SMP – colic, constipation	> 0
2	A	SMP – colic, constipation	> 0
3	A	SMP – regurgitation	0 – 6
4	A	SMP – regurgitation	0 – 6
5	A	SMP – regurgitation	0 – 6
6	A	PIF (tabs)	0 – 6
7	A	PIF (tabs)	0 – 6
8	A	FOF (tabs)	6 – 12
9	A	FOF (tabs)	6 – 12
10	A	FOF	> 12
11	A	FOF	> 12
12	A	FOF	> 24
13	A	FOF	> 24
14	A	FOF	> 24
15	A	PIF	0 – 6
16	A	PIF	0 – 6
17	A	FOF	6 – 12
18	A	FOF	6 – 12
19	A	FOF	> 12
20	A	FOF	> 12
21	B	SMP – allergy	0 – 12
22	B	SMP – allergy	12 – 32
23	B	SMP – allergy	12 – 32
24	B	SMP – colic, constipation	> 0
25	B	SMP – colic, constipation	> 0
26	B	SMP – regurgitation	> 0
27	B	SMP – regurgitation	> 0
28	B	PIF	0 – 12
29	B	PIF	0 – 12
30	B	FOF	12 – 36
31	C	PIF	0 – 6
32	C	PIF	0 – 6
33	C	FOF	> 6
34	C	FOF	> 6
35	C	FOF	12 – 36
36	C	FOF	12 – 36
37	C	PIF	0 – 6
38	D	PIF	0 – 6
39	D	PIF	0 – 6
40	D	FOF	6 – 12
41	D	FOF	6 – 12

42	D	FOF	> 12
43	D	FOF	> 12
44	D	FOF	> 24
45	D	FOF	> 24
46	E	SMP – colic, constipation	> 0
47	E	SMP – regurgitation	0 – 6
48	E	SMP – regurgitation	0 – 6
49	E	SMP – regurgitation	6 – 12
50	E	SMP – regurgitation	6 – 12
51	E	PIF	0 – 6
52	E	PIF	0 – 6
53	E	FOF	6 – 12
54	E	FOF	6 – 12
55	E	FOF	12 – 24
56	E	FOF	12 – 24
57	E	FOF	24 – 36
58	E	FOF	24 – 36
59	E	PIF	0 – 6
60	E	FOF	6 – 12
61	F	PIF	> 0
62	F	FOF	> 6
63	F	FOF	> 6
64	G	PIF	> 0
65	G	FOF	> 6
66	G	FOF	> 6
67	G	PIF	0 – 6
68	H	SMP – colic, constipation	> 0
69	H	PIF	0 – 6
70	H	PIF	0 – 6
71	H	FOF	6 – 12
72	H	FOF	12 – 24
73	I	PIF	> 0
74	I	PIF	> 0
75	I	PIF	> 0
76	I	FOF	> 6
77	I	FOF	> 6
78	I	FOF	> 6
79	I	FOF	12 – 36
80	I	FOF	12 – 36
81	J	PIF	0 – 6
82	J	FOF	6 – 12
83	J	FOF	12 – 36

Supplementary file S2

Microbial characterization and isolates identification

LOD= 2 Log CFU/g except for B. cereus (LOD= 1 Log CFU/g)

#	Total mesophilic bacteria	N. colonies isolated and identification	Anaerobic bacteria	N. colonies isolated and identification	LAB	N. colonies isolated and identification	Molds	N. colonies isolated and identification	Bacillus cereus P/A	N. colonies isolated and identification	Bacillus cereus enumeration	N. colonies isolated and identification
1	<LOD		<LOD		<LOD		<LOD					
2	<LOD		<LOD		<LOD		<LOD					
3	<LOD		<LOD		<LOD		<LOD		P	*1. B. cereus*		
4	2.30	*1. Klebsiella aerogenes*	<LOD		<LOD		<LOD					
5	2.48	*1. Weizmannia ginsengihumi*	<LOD		<LOD		<LOD					
6	<LOD		<LOD		<LOD		<LOD		P	*1. B. cereus*		
7	<LOD		<LOD		<LOD		<LOD		P	*2. B. cereus*		
8	<LOD		<LOD		<LOD		<LOD					
9	<LOD		<LOD		<LOD		<LOD		P	*1. B. cereus*		
10	<LOD		<LOD		<LOD		1.00	*1. Penicillum sp.*	P	*1. B. cereus*		
11	<LOD		<LOD		<LOD		<LOD					
12	<LOD		2.48	*2. Moraxella osloensis*	<LOD		<LOD		P	*1. B. cereus*		
13	<LOD		<LOD		<LOD		<LOD					
14	<LOD		<LOD		<LOD		<LOD		P	*1. B. cereus*		
15	<LOD		<LOD		<LOD		<LOD					
16	<LOD		<LOD		<LOD		<LOD					
17	<LOD		<LOD		<LOD		<LOD		P	*1. B. cereus*		
18	<LOD		<LOD		<LOD		<LOD		P	*1. B. cereus*		
19	<LOD		<LOD		<LOD		<LOD					
20	<LOD		<LOD		<LOD		<LOD		P	*1. B. cereus/thuringensis*		
21	<LOD		<LOD		<LOD		1.00					
22	<LOD		<LOD		<LOD		1.00		P	*1. B. cereus/thuringensis*		
23	<LOD		<LOD		<LOD		<LOD		P	*1. B. cereus/thuringensis*		
24	<LOD		<LOD		<LOD		<LOD		P	*1. B. cereus/thuringensis*		
25	<LOD		<LOD		<LOD		<LOD					
26	<LOD		<LOD		<LOD		<LOD					
27	2.30	*1. Bacillus subtilis; 1. Bacillus sonnolensis*	<LOD		<LOD		<LOD					
28	<LOD		<LOD		<LOD		1.00					
29	<LOD		<LOD		<LOD		<LOD					
30	<LOD		<LOD		<LOD		<LOD					
31	6.46	*5. Limosilactobacillus fermentum*	6.61	*5. Limosilactobacillus fermentum*	6.73	*5. Limosilactobacillus fermentum*	<LOD					
32	6.40	*5. Limosilactobacillus fermentum*	6.65	*5. Limosilactobacillus fermentum*	6.61	*3. Limosilactobacillus fermentum*	<LOD					
33	<LOD		6.29	*5. Limosilactobacillus fermentum*	6.39	*3. Limosilactobacillus fermentum*	<LOD		P	*1. B. cereus*		

34	7.26	*5. Limosilactobacillus fermentum*	7.27	*5. Limosilactobacillus fermentum*	7.37	*3. Limosilactobacillus fermentum*	<LOD					
35	<LOD		6.45	*5. Limosilactobacillus fermentum*	6.58	*3. Limosilactobacillus fermentum*	<LOD		P	*2. B. cereus*		
36	<LOD		6.18	*5. Limosilactobacillus fermentum*	6.40	*5. Limosilactobacillus fermentum*	<LOD		P	*1. B. cereus*		
37	<LOD		<LOD		6.61	*3. Limosilactobacillus fermentum*	<LOD		P	*1. B. cereus/thuringensis*		
38	<LOD		1.30		<LOD		<LOD					
39	<LOD		<LOD		<LOD		<LOD		P	*1. B. cereus*		
40	<LOD		<LOD		<LOD		<LOD		P	*1. B. cereus*		
41	<LOD		<LOD		<LOD		<LOD		P	*1. B. cereus*		
42	<LOD		<LOD		<LOD		<LOD		P	*1. B. cereus*		
43	<LOD		2.30		<LOD		<LOD		P	*1. B. cereus/thuringensis*		
44	<LOD		<LOD		<LOD		<LOD					
45	<LOD		<LOD		<LOD		<LOD					
46	<LOD		<LOD		<LOD		<LOD					
47	<LOD		<LOD		<LOD		<LOD					
48	<LOD		<LOD		<LOD		<LOD					
49	<LOD		<LOD		<LOD		<LOD		P	*1. B. cereus/thuringensis*	1.00	*1. B. cereus/thuringensis*
50	<LOD		<LOD		<LOD		<LOD					
51	<LOD		4.24		<LOD		<LOD					
52	3.50	*1. Staphylococcus warneri*	3.82		<LOD		<LOD					
53	<LOD		3.88		<LOD		<LOD					
54	<LOD		<LOD		<LOD		<LOD		P	*1. B. cereus/thuringensis*		
55	<LOD		<LOD		<LOD		<LOD		P	*1. B. cereus*		
56	2.00	*1. Bacillus licheniformis*	<LOD		<LOD		<LOD					
57	<LOD		<LOD		<LOD		1.00	*1. Penicillum sp.*	P	*1. B. cereus*		
58	<LOD		<LOD		<LOD		1.00	*1. Penicillum sp.*	P	*1. B. cereus*		
59	<LOD		<LOD		<LOD		<LOD					
60	<LOD		<LOD		<LOD		<LOD					
61	<LOD		<LOD		<LOD		<LOD					
62	<LOD		<LOD		<LOD		<LOD					
63	<LOD		<LOD		<LOD		<LOD					
64	<LOD		<LOD		6.06	*3. Limosilactobacillus reuteri*	<LOD		P	*1. B. cereus*		
65	2.30	*1. Bacillus licheniformis*	<LOD		6.14	*3. Limosilactobacillus reuteri*	<LOD		P	*1. B. cereus*		
66	<LOD		<LOD		6.14	*3. Limosilactobacillus reuteri*	<LOD		P	*1. B. cereus*		
67	<LOD		<LOD		<LOD		1.00					
68	<LOD		<LOD		<LOD		<LOD		P	*1. B. cereus/thuringensis*		
69	<LOD		<LOD		<LOD		<LOD					
70	<LOD		<LOD		<LOD		4.01	*1. Penicillum sp.*				
71	<LOD		<LOD		<LOD		<LOD					
72	<LOD		<LOD		<LOD		<LOD					
73	<LOD		<LOD		<LOD		<LOD		P	*1. B. cereus*		
74	<LOD		<LOD		<LOD		<LOD		P	*1. B. cereus*	1.00	
75	<LOD		<LOD		<LOD		<LOD		P	*1. B. cereus/thuringensis*	1.00	

76	2.00	*1. Bacillus subtilis*	<LOD		<LOD		<LOD					
77	<LOD		<LOD		<LOD		<LOD		P	*1. B. cereus*		
78	<LOD		<LOD		<LOD		<LOD		P	*1. B. cereus/thuringensis*		
79	<LOD		<LOD		<LOD		<LOD		P	*1. B. cereus*		
80	<LOD		<LOD		<LOD		1.00					
81	<LOD		<LOD		<LOD		<LOD					
82	<LOD		<LOD		<LOD		<LOD					
83	<LOD		<LOD		<LOD		<LOD					

P=present

Supplementary file S3

Chemical-physical characterization

#	pH	Aw	Moisture (%)	SS (%)
1	6.69	0.2058	2.92	97.08
2	6.66	0.2366	2.84	97.16
3	6.35	0.3234	3.45	96.55
4	6.42	0.3211	3.41	96.59
5	6.36	0.3173	3.14	96.86
6	6.78	0.2997	3.09	96.91
7	6.56	0.2950	2.69	97.31
8	6.60	0.2901	2.35	97.65
9	6.58	0.2586	2.09	97.91
10	6.82	0.2207	2.12	97.88
11	6.73	0.1586	1.98	98.02
12	6.71	0.2459	2.45	97.55
13	6.73	0.2165	2.28	97.72
14	6.60	0.2254	2.29	97.71
15	6.52	0.3051	2.81	97.19
16	6.62	0.2965	2.91	97.09
17	6.60	0.3017	2.88	97.12
18	6.50	0.3127	2.92	97.08
19	6.76	0.2834	3.10	96.90
20	6.75	0.1755	2.59	97.41
21	6.21	0.2293	1.94	98.06
22	6.02	0.1858	2.54	97.46
23	5.86	0.1428	2.66	97.34
24	5.80	0.2006	2.57	97.43
25	5.89	0.1637	2.72	97.28
26	6.58	0.1845	2.30	97.70
27	6.52	0.1705	2.01	97.99
28	6.69	0.1459	1.41	98.59
29	6.70	0.1397	2.34	97.66
30	6.59	0.1855	1.89	98.11
31	6.44	0.2856	3.02	96.98
32	6.43	0.2916	3.02	96.98
33	6.62	0.2841	2.47	97.53
34	6.68	0.2997	2.74	97.26
35	7.24	0.3196	2.90	97.10
36	7.17	0.3160	2.73	97.27
37	6.48	0.2732	2.01	97.99
38	6.92	0.2868	2.56	97.44
39	7.04	0.1609	2.16	97.84
40	6.95	0.2779	2.53	97.47
41	7.01	0.1438	2.13	97.87
42	6.67	0.3037	2.37	97.63
43	6.75	0.2747	2.54	97.46

44	6.68	0.3121	2.86	97.14
45	6.80	0.1495	2.05	97.95
46	6.74	0.1812	2.20	97.80
47	6.35	0.1848	2.15	97.85
48	6.34	0.1825	2.15	97.85
49	6.31	0.2093	2.51	97.49
50	6.31	0.2017	2.39	97.61
51	6.42	0.2564	2.22	97.78
52	6.52	0.2653	2.06	97.94
53	6.50	0.2323	2.05	97.95
54	6.40	0.1826	1.62	98.38
55	6.71	0.2560	2.38	97.62
56	6.82	0.1947	1.73	98.27
57	6.82	0.1892	1.59	98.41
58	6.77	0.1776	1.67	98.33
59	5.89	0.1377	2.43	97.57
60	5.97	0.2080	2.22	97.78
61	6.12	0.2163	1.73	98.27
62	6.57	0.2070	1.69	98.31
63	6.60	0.2028	1.62	98.38
64	6.60	0.1996	1.30	98.70
65	6.42	0.2099	1.28	98.72
66	6.52	0.2051	1.21	98.79
67	6.89	0.1912	2.43	97.57
68	6.62	0.2028	2.26	97.74
69	6.60	0.3166	1.92	98.08
70	6.60	0.2743	1.96	98.04
71	6.59	0.2106	1.77	98.23
72	6.67	0.1996	1.79	98.21
73	6.33	0.1327	0.94	99.06
74	6.32	0.1377	1.42	98.58
75	6.35	0.1459	1.20	98.80
76	6.38	0.1831	1.95	98.05
77	6.42	0.1530	1.74	98.26
78	6.42	0.1252	1.80	98.20
79	6.38	0.1789	2.05	97.95
80	6.45	0.1301	1.38	98.62
81	6.82	0.1341	2.38	97.62
82	6.93	0.1755	2.23	97.77
83	6.82	0.1578	2.47	97.53
